# The *N*-linking glycosylation system from *Actinobacillus pleuropneumoniae* is required for adhesion and has potential use in glycoengineering

**DOI:** 10.1098/rsob.160212

**Published:** 2017-01-11

**Authors:** Jon Cuccui, Vanessa S. Terra, Janine T. Bossé, Andreas Naegeli, Sherif Abouelhadid, Yanwen Li, Chia-Wei Lin, Prerna Vohra, Alexander W. Tucker, Andrew N. Rycroft, Duncan J. Maskell, Markus Aebi, Paul R. Langford, Brendan W. Wren, on behalf of the BRaDP1T Consortium

**Affiliations:** 1 Faculty of Infectious and Tropical Diseases, London School of Hygiene and Tropical Medicine, Keppel Street, London WC1E 7HT, UK; 2 Section of Paediatrics, Department of Medicine, Imperial College London, St. Mary's Campus, London W2 1PG, UK; 3 Institute of Microbiology, ETH Zürich, Wolfgang-Pauli-Strasse 10, 8093 Zürich, Switzerland; 4 Department of Veterinary Medicine, University of Cambridge, Madingley Road, Cambridge CB3 0ES, UK; 5 The Royal Veterinary College, Hawkshead Campus, Hatfield, Hertfordshire AL9 7TA, UK

**Keywords:** *N*-linked glycosylation, *Actinobacillus pleuropneumoniae*, adhesion

## Abstract

*Actinobacillus pleuropneumoniae* is a mucosal respiratory pathogen causing contagious porcine pleuropneumonia. Pathogenesis studies have demonstrated a major role for the capsule, exotoxins and outer membrane proteins. *Actinobacillus pleuropneumoniae* can also glycosylate proteins, using a cytoplasmic *N*-linked glycosylating enzyme designated NGT, but its transcriptional arrangement and role in virulence remains unknown. We investigated the NGT locus and demonstrated that the putative transcriptional unit consists of *rimO*, *ngt* and a glycosyltransferase termed *agt.* From this information we used the *A. pleuropneumoniae* glycosylation locus to decorate an acceptor protein, within *Escherichia coli,* with a hexose polymer that reacted with an anti-dextran antibody. Mass spectrometry analysis of a truncated protein revealed that this operon could add up to 29 repeat units to the appropriate sequon. We demonstrated the importance of NGT in virulence, by creating deletion mutants and testing them in a novel respiratory cell line adhesion model. This study demonstrates the importance of the NGT glycosylation system for pathogenesis and its potential biotechnological application for glycoengineering.

## 1. Introduction

*Actinobacillus pleuropneumoniae* is a Gram-negative bacterium and the causative agent of porcine pleuropneumonia, a severe respiratory disease responsible for significant losses to the pig industry worldwide. Economically, this disease has a huge impact on the pig industry, costing an average €6.4 per fattened pig in an affected herd in Europe [1]. *Actinobacillus pleuropneumoniae* enters the lungs and colonizes tissues by binding to mucus proteins and cells of the lower respiratory tract, including ciliated cells of the terminal bronchioli and alveolar epithelial cells [2,3]. There are 15 established serovars that differ in capsular polysaccharide composition [4], with another proposed based on serological results [5]. Several surface structures have been identified as being involved in adhesion, including fimbriae [6] and lipopolysaccharide (LPS) [7].

Advances in DNA sequencing technologies and mass spectrometry techniques reveal that post-translational modification of proteins by glycosylation is not restricted to a few bacterial species and is often important in pathogenesis [8,9]. Understanding the mechanisms of bacterial glycosylation and its role in pathogenesis can have practical applications such as the design of novel bioglycoconjugate vaccines, antimicrobials and diagnostics [10,11]. Bacterial protein glycosylation systems can be broadly divided into two main categories: glycans that are covalently attached to amide groups of asparagine residues (*N*-linked) or to hydroxyl groups on serine/threonine residues (*O*-linked). These categories can be further subdivided depending on the cellular compartment where protein glycosylation takes place. Oligosaccharyltransferases (OTases) function in the periplasmic compartment of a bacterial cell and catalyse the transfer of an oligosaccharide from a lipid donor to an acceptor molecule, usually a protein. The best-studied bacterial OTases are the *C. jejuni* PglB system, where *en bloc* glycosylation operates through an *N*-OTase [12,13], and the *Neisseria meningitidis O*-OTase PglL. *N*- and *O*-linked glycosylation can also occur in the cytoplasmic compartment of the bacterial cell, mediated through the action of glycosyltransferases that use nucleotide activated sugar donors as substrates for transfer onto the acceptor protein. Examples of cytoplasmic glycosylation can be found in *Clostridium difficile*, where flagellin is *O*-glycosylated [14], and non-type-able *Haemophilus influenzae* (from here on referred to as NTHi) [15], where two copies of a cytoplasmic *N*-linked glycosylation modify a high-molecular-weight adhesin with hexoses using the enzyme HMWC. In NTHi*,* all proteins responsible for high-molecular-weight adhesin synthesis, transport and glycosylation are encoded in the same locus.

**Figure 1.**
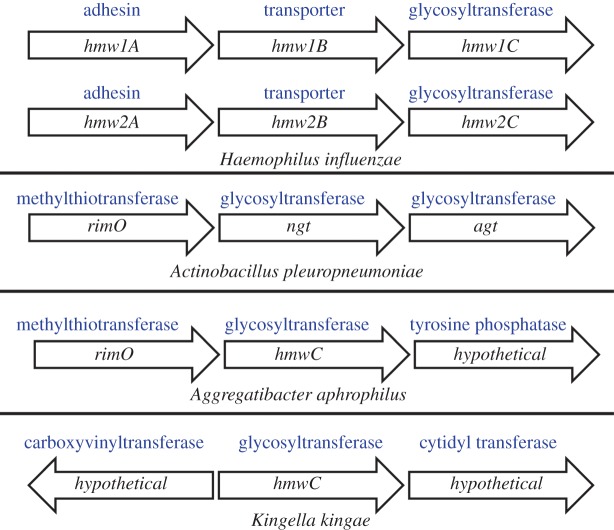
Genetic organization of the HMWC enzyme family. In contrast to other bacteria, NTHi has two copies of the HMW locus, and each has a gene encoding an acceptor protein. *A. pleuropneumoniae* is the only species that has a second glycosyltransferase adjacent to the *N*-linking enzyme.

*Actinobacillus pleuropneumoniae* also carries a cytoplasmic *N*-linking glycosyltransferase, known as NGT. It is a member of the HMWC-like glycosyltransferase family [16–20], but lacks an adjacent adhesin or transporter and its transcriptional unit remains to be characterized. Recently, studies into the human pathogens *Kingella kingae* and *Aggregatibacter aphrophilus* [21] demonstrated a similar genetic arrangement. These ‘orphan’ HMWC enzymes have been found to glycosylate trimeric autotransporter adhesins, encoded in distant locations of the genome [21]. Autotransporter proteins, such as the trimeric autotransporter adhesin (TAA) Apa found in *A. pleuropneumoniae*, mediate attachment to host cells [22]. Apa is predicted to have an *N*-terminal signal peptide for secretion, a functional passenger domain containing head, neck and stalk motifs, and a conserved C-terminal translocator domain [22]. However, *A. pleuropneumoniae* has a unique chromosomal feature. Adjacent to *ngt*, there is a second ORF, which we named *agt,* coding for an accessory glycosyltransferase (figure 1).

When *agt* is heterologously expressed in *Escherichia coli* and purified, it can be used *in vitro,* to add further glucose residues to the *N*-linked glycan that NGT generates [19]. However, *agt* has never been demonstrated to function *in vivo* in conjunction with *ngt*. In addition, no virulence phenotype has been reported in *A. pleuropneumoniae* for this glycosylation locus owing to known difficulties in constructing genetic mutations in this organism.

In this study, we report the generation of *A. pleuropneumoniae ngt* and *agt* deletion mutants, and demonstrate a biological role for this *N*-linked glycosylation system using a human adenocarcinoma lung epithelial cell adhesion assay. Our results suggest that *ngt* is part of an operon that contains the upstream ORF *rimO*, encoding a methylthiotransferase, and the downstream ORF *agt*, encoding an α-6-glucosyltransferase (α6GlcT). Furthermore, we were able to clone and express *ngt* and *agt* in *E. coli*, demonstrating for the first time, to the best of our knowledge, the *in vivo* assembly of *N*-linked dextran.

## 2. Material and methods

### 2.1. Bacterial strains used and culture conditions

*Actinobacillus pleuropneumoniae* serovar 15 reference strain, HS143, or derived mutants were grown at 37°C with 5% CO_2_ on BHI (Oxoid, UK) agar or broth, supplemented with 10 µg ml^−1^ nicotinamide adenine dinucleotide (NAD) and when required with kanamycin (50 µg ml^−1^) or chloramphenicol (1 µg ml^−1^). *Escherichia coli* TOP10 and Mu Free Donor (MFD) [23] were grown in LB broth or agar (Oxoid) supplemented, when required, with 50 µg ml^−1^ kanamycin at 37°C. *E. coli* DH10 were grown in LB broth or agar (Oxoid) at 37°C supplemented, when required, with 80 µg ml^−1^ spectinomycin and/or 100 µg ml^−1^ trimethoprim.

### 2.2. Genomic DNA extraction

Total genomic DNA was extracted from a 10 ml overnight culture of *A. pleuropneumoniae* HS143, using a proteinase K and phenol: chloroform: isoamyl-alcohol-based procedure as previously described by Cuccui *et al.* [24].

### 2.3. Construction of *Actinobacillus pleuropneumoniae* knockout mutants

The *A. pleuropneumoniae* HS143 orthologues of *apl_1634* and *apl_1635* (also known as *agt* and *ngt*, coding for α6GlcT and NGT, respectively), found in the *A. pleuropneumoniae* L20 genome [25], were deleted using our recently described unmarked mutation system [26]. Primers used to generate the *cat-sacB* insertion/deletion and the unmarked deletion constructs for each gene are shown in electronic supplementary material, table S2. Briefly, the target genes and approximately 600–900 bp of flanking sequences were amplified using CloneAmp HiFi PCR Premix (Clontech), A-tailed and cloned into pGEMT (Promega), as previously described [26]. Inverse PCR was then used to open up the clones, using the appropriate primers, removing the target sequence and adding 15 bp overhangs to allow insertion of the *cat-sacB* cassette by In-Fusion cloning (Clontech). Unmarked deletion constructs were generated by amplifying the left and right flanking sequences for each gene, using appropriate primers with added 15 bp overhangs designed to allow direct fusion by overlap-extension PCR. The unmarked deletion mutants were then obtained by two sequential rounds of natural transformation as previously described [27].

### 2.4. Plasmid complementation

The vector pMKExpress [28] was digested with *Eco*RI and *Sac*I (New England Biolabs, UK) and the resulting digest was gel purified using a Qiagen MinElute gel extraction kit (Qiagen, UK) according to the manufacturer's instructions, to remove the GFP coding ORF.

The *ngt* gene was PCR amplified using Accuprime Taq Hifi (Invitrogen, UK) using the forward primer ngtCOMPFWD (5′-TTTTGAATTCGTGGGTAAAACGCTTGCAGT-3′) and reverse primer ngtCOMPREV (5′-TTTTGAGCTCTTAATTTTCTTTTAGGAACGCATTT-3′). The *agt* gene was amplified using the primers agtCOMPFWD (5′-AAACTGCAGATTAAATGCGTTCCTAAAAGAAAA-3′) and agtCOMPREV (5′-TTTGCGGCCGCTTAACTCCGACTATTCTCAAG-3′).

When *agt* only complementation failed, complementation with *ngt–agt* was attempted. Both ORFs were PCR amplified using Accuprime Taq Hifi (Invitrogen) using the forward primer ngtagtCOMPFWD (5′-TTTGAATTCCGAGCAAGAAGTGAAAGTCG-3′) and reverse primer ngtagtCOMPREV (5′-TTTGCGGCCGCCACCGATAGCCGTATTTCGT-3′) with the following cycling conditions: 94°C/30 s followed by 24 cycles of 94°C/30 s, 53°C/30 s, 68°C/2 min and a final 68°C/5 min cycle. All ORFS were expressed under the control of the plasmid promoter.

The resulting *ngt* only PCR product was digested with *Eco*RI and *Sac*I, the *agt* only product was digested with *Pst*I and *Not*I, and the *ngt–agt* PCR product was digested with *Eco*RI and *Not*I before being purified using a Qiagen PCR purification kit. Digested vector and PCR products were ligated using Promega T4 DNA ligase (Promega, UK) to yield the vectors pMK*ngt,* pMK*agt* and pMK*ngt*–*agt,* prior to transformation of the plasmid into One Shot *E. coli* TOP10 cells (Invitrogen) according to manufacturer's instructions. Transformants were selected on LB agar supplemented with kanamycin (50 µg ml^−1^). The complementation vectors were transformed into the mutant recipient strains by natural transformation as previously described [27].

### 2.5. Cell culture

The A549 cell line, adenocarcinoma human alveolar basal epithelial cells, (ATCC, CCL-185, US) was grown at 37°C, 5% CO_2_ in F-12 K medium (Gibco) supplemented with 10% fetal calf serum (Sigma).

### 2.6. Adhesion assay using A549 cell line

The A549 cell line (ATCC, CCL-185, USA) was seeded into 12-well tissue culture plates at a concentration of 2.5 × 10^5^ cells ml^−1^ and incubated overnight at 37°C 5% CO_2_. Bacterial overnight cultures (HS143 wild-type, isogenic *ngt* and *agt* mutants and complemented mutants) were used to seed into BHI–NAD medium and grown to an OD_600 nm_ of 0.6. One millilitre of the suspension was added to the A549 cells at a multiplicity of infection (MOI) of 100 : 1, and the plates incubated at 37°C 5% CO_2_. After 3 h, non-adherent bacteria were removed by washing three times with 1 ml DPBS (Gibco), and adherent bacteria were released by adding 100 µl of 0.25% trypsin–EDTA (Sigma) for 5 min at 37°C. Trypsinization was stopped by the addition of 900 µl of DPBS. Serial dilutions were plated onto BHI–NAD plates for quantification of adherent bacteria. In order to determine if any of the recovered bacteria had invaded the A549 cells, controls were treated with gentamycin (Sigma) at a final concentration of 10 µg ml^−1^ for 1 h to allow for killing of adherent extracellular bacteria. The cells were then lysed by the addition of ice-cold sterile water, and serial dilutions were plated out on BHI–NAD.

### 2.7. Statistical analysis of adhesion assay data

The number of adherent cells was calculated by counting the colony forming units and comparing with the initial inoculum of each individual culture to determine the percentage of adherent cells. The statistical analysis was performed using a one-way analysis of variance followed by a Bonferroni's multiple comparison test. The significance level was set at 0.05 throughout. Statistical analysis was done using GraphPad Prism v. 4.00 for Windows (GraphPad Software, San Diego, CA, www.graphpad.com).

### 2.8. Reverse transcriptase PCR

An overnight culture of *A. pleuropneumoniae* HS143 was diluted 1 : 20 in BHI–NAD broth. At the time points of 1.5, 3.0, 5 and 24 h, RNA was extracted as previously described [29], with the minor modification that 2 µg of total RNA from each sample was treated with Ambion TURBO DNase (Invitrogen) according to the manufacturer's instructions. cDNA was generated from DNase-treated RNA using the SuperScript II kit (Invitrogen) according to the manufacturer's instructions. For each sample, 2 µl of reverse transcribed cDNA was used as a template in a 25 µl total volume PCR mixture and amplified using MyTaq master mix (Bioline, UK) using the following cycling conditions: 94°C/30 s, followed by 35 cycles of 94°C/10 s, 53°C/10 s, 72°C/10 s and a final single 72°C/30 s cycle using the primers listed in electronic supplementary material, table S2.

### 2.9. Quantitative real-time PCR validation

Total RNA was extracted from bacteria grown in BHI–NAD broth by using Tri Reagent (Sigma, UK) as previously described. Two micrograms from each sample was treated with Ambion TURBO DNase (Invitrogen) according to manufacturer's instructions with some modifications. Total RNA was incubated for 1 h at 37°C followed by the addition of another two units of DNase. The sample was then incubated for an extra 1 h before inactivation.

cDNA was generated from DNase-treated RNA using the SuperScript III kit (Invitrogen) using random hexamers (Invitrogen) according to the manufacturer's instructions. One microlitre of this material was used in a QPCR using SYBR Green dye-based PCR amplification and detection system (Applied Biosystems). The *A. pleuropneumoniae* HS143 WT, HS143Δ*ngt* and HS143Δ*agt* were analysed for absolute quantification of cDNA using an ABI7500 Fast instrument (Applied Biosystems). Amplification was carried out using the following primers at a final concentration of 500 nM. agtfwd: 5′-GAT TGG ATA GGT GAA GGC GA-3′, agtrev: 5′-CCC TTG CTC AAA ATG ACG GA-3′, ngtfwd: 5′-AGT TTG TGA GAG CAA CGG TG-3′, ngtrev: 5′-AGT CCG AAT GTG TTG TTG CC-3′, rimOfwdv2: 5′-CGT CCG ATT GTG CAA GTG TT-3′, rimOrevv2: 5′-CAC CGT TCC AGA AAA CCG TT-3′. Samples tested were four biological replicates, each tested as three technical replicates.

For comparative qRTPCR analysis of *A. pleuropneumoniae* HS143 WT, HS143Δ*ngt* and HS143Δ*agt*, gene induction or reduction values were calculated by comparing the normalized values of the wild-type and mutant samples, using the statistical formulation for the threshold cycle (ΔΔCT) method. The threshold value of each gene was first normalized to the value of the constitutively expressed control gene *glyA* [30] (glyA primers: fwd: 5′-CAA GCG AAT GCA GCT GTT TA-3′, glyArev: 5′- CTG TGA TGC CGT AGA GGA CA-3′).

### 2.10. Subcloning and heterologous expression of *agt* and *ngt*

The putative NGT operon was PCR amplified using the primers *ngt*-*agt*fwd: 5′-TTTTGAATTCCGAGCAAGAAGTGAAAGTCG-3′ and *ngt-agt*rev: 5′-TTTTTGGTACCCACCGATAGCCGTATTTCGT-3′ using Accuprime Taq Hifi (Invitrogen) and the following cycling conditions: 94°C/30 s, followed by 24 cycles of 94°C/30 s, 53°C/30 s, 68°C/4 min and a final cycle of 68°C/5 min.

The amplicon was ligated into the vector pEXT20 using T4 DNA ligase (New England Biolabs, UK) following digestion of the plasmid and the PCR product with *Eco*RI and *Kpn*I. *Escherichia coli* NEB10β (New England Biolabs, UK) was transformed with the ligation reaction generating the plasmid pJC78. Expression was induced by growing an *E. coli* colony in LB broth with ampicillin 100 µg ml^−1^ until an OD_600_ of 0.4 was reached. At that point 1 mM, IPTG was added, and the cultures were incubated at 37°C with shaking for a further 16 h. Expression of NGT and α6GlcT was monitored using SDS–PAGE, Coomassie staining and western blotting.

### 2.11. Glycosylation of AtaC by NGT and α6GlcT in *Escherichia coli* cells

*Escherichia coli* DH10β cells carrying pJC78 were transformed with the construct pMLBADAtaC_1866–2428_ [17], and cultured in LB broth with ampicillin 100 µg ml^−1^, trimethoprim 20 µg ml^−1^ at 37°C with shaking until an OD_600 nm_ of 0.4 was reached, followed by induction with 0.2% l-arabinose and 1 mM IPTG. After 16 h incubation, AtaC was purified. The bacterial cell pellet was isolated by centrifugation at 6000*g* for 10 min and lysed using a cell homogenizer (Stansted Fluidics Ltd. SPCH-10). Any intact cell debris was thereafter pelleted by centrifugation at 10 000*g* for 30 min before purification from the supernatant using an Ni–NTA (Qiagen, UK) gravity column (Thermo Scientific, USA).

Glycosylated product was analysed by SDS–PAGE and transferred onto a nitrocellulose membrane before being analysed by immunoblot using a mouse anti-His antibody (AbCam, UK) and an IRDye 680CW goat anti-mouse conjugate secondary antibody. Detection of fluorescent signal was carried out using a LI-COR imaging system.

### 2.12. Mutagenesis of the *ngt* locus

The cloned locus coding for NGT and α6GlcT was mutated using the QuickChange XL II site-directed mutagenesis kit (Agilent Technologies, CA) using the following primers. agtt120a_antis: 5′-CAAAACAGAAGTAAACGTTTTAATCTATATTATTTTCCATAACAT AACCTTAAGAGCC-3′ and agtt120a: 5′-GGCTCTTAAGGTTATGTTATGGAAAATAAT ATAGATTAAAACGTTTACTTCTGTTTTG-3′ (underlined nucleotide denotes the change).

The *ngt* gene was mutated using the following primers: ngta1321g_a132: 5′-CGGTATAGCTTCAACCACGATGGCGCTAAATCCGTATTT CTTAGAA-3′ and ngta1321g_a132: 5′-TTCTAAGAAATACGGATTTAGCGCCATCGTGGTTGAAGCTATACCG-3′ (underlined nucleotides denotes the change). The following conditions were used: 95°C/60 s followed by 18 cycles of 95°C/50 s, 60°C/50 s, 68°C/8 min and a final 68°C/7 min cycle.

Following amplification, the PCR products were *Dpn*I treated according to the manufacturer's instructions and used to transform *E. coli* XL-10 Gold cells (New England Biolabs, UK).

### 2.13. Western blot analysis of glycosylated AtaC

Purified AtaC from *E. coli* DH10β was analysed by western blotting. Unglycosylated, fully glycosylated and monoglycosylated AtaC were analysed by dot blot by placing a 3 µl drop of a 1 mg ml^−1^ solution of protein or dextran (dextran standard MW 1000 from Leuconostoc, Sigma-Aldrich UK) onto a nitrocellulose blotting membrane (Amersham Protran, GE HealthCare, Germany) and allowing to air dry before blocking the membrane by incubating with phosphate-buffered saline, 2% milk solution for 1 h at room temperature. The membrane was then probed using a 1 : 1000 dilution of a mouse monoclonal antibody raised specifically against a tetrasaccharide of α1–6 linked glucose (MS α-Dextran Clone Dx1, Stem Cell Technologies, Canada). An IRDye 680CW goat anti-mouse antibody at a 1 : 10 000 dilution was used as the secondary antibody. The western blot images were visualized using a LI-COR imaging system.

### 2.14. Analysis of N-glycans released from AtaC

Glycans were released from 200 µg AtaC using the Ludger Liberate Hydrazinolysis kit, according to the manufacturer's recommendations. The released glycans were fluorescently labelled with 2-aminobenzamide (2-AB) as described previously [31]. Excess labelling reagent was removed as follows: four discs of filter paper (Whatman) were soaked in 30% acetic acid, inserted into a 1 ml plastic syringe and washed sequentially with 2 × 1 ml acetic acid, 2 × 1 ml water, 2 × 1 ml acetonitrile (ACN) and finally 2 × 1 ml 95% ACN. The labelled glycans were diluted to 500 µl with 95% ACN and loaded onto the column. Of 500 µl 95% ACN was used to rinse the labelling tube and was added onto the column as well. The column was washed with 8 × 1 ml 95% ACN and glycans were finally eluted in 50 µl water twice. Elution fractions were pooled and passed through a 0.45 µm filter (Ultrafree-MC Durapore HV filter unit, Millipore) before analysis by normal-phase HPLC (Supelcosil LC-NH2 column, 80–20% ACN gradient over 90 min, fluorescence detection at 320 nm excitation and 420 nm emission wavelength). Glyko 2-AB glucose homopolymer standard (Prozyme) was used as a reference. The identity of the labelled glycans was confirmed by MALDI mass spectrometry. Samples were mixed 1 : 1 with dihydroxybenzonic acid matrix (15 mg ml^−1^ in 75% ACN in water with 0.1% formic acid (FA)), and spotted onto a matrix-assisted laser desorption/ionization time of flight mass spectrometry (MALDI-TOF–TOF MS) target plate. Data acquisition was performed manually on a Model 4800 Proteomics Analyser (Applied Biosystems, Framingham, MA) with an Nd : YAG laser, and 1000 shots were accumulated in the reflectron positive ion mode.

### 2.15. Nano-LC–ESI–MS/MS analysis of glycosylated AtaC

For structural analysis, 50 µg of AtaC was reduced, alkylated and digested with trypsin using the filter-aided sample preparation protocol [32]. Samples were analysed on a calibrated LTQ-Orbitrap Velos mass spectrometer (Thermo Fischer Scientific, Bremen, Germany) coupled to an Eksigent-Nano-HPLC system (Eksigent Technologies, Dublin (CA)). Peptides were resuspended in 2.5% ACN and 0.1% FA, and loaded on a self-made tip column (75 µm × 80 mm) packed with reverse phase C18 material (AQ, 3 µm 200 Å, Bischoff GmbH, Leonberg, Germany) and eluted with a flow rate of 200 nl per min by a gradient from 3% to 30% ACN, 0.1% FA in 22 min, 50% ACN, 0.1% FA in 25 min, 97% ACN, 0.1% FA in 27 min. One scan cycle comprised a full-scan MS survey spectrum, followed by up to 20 sequential collision-induced dissociation (CID) MS/MS on the most intense signals above a threshold of 1500. Full-scan MS spectra (400–2000 *m*/*z*) were acquired in the FT-Orbitrap at a resolution of 60 000 at 400 *m*/*z*, whereas CID MS/MS spectra were recorded in the linear ion trap. CID was performed with a target value of 1e4 in the linear trap, collision energy at 35 V, *Q*-value at 0.25 and activation time at 30 min. AGC target values were 5e5 for full FTMS scans and 1e4 for ion trap MSn scans. For all experiments, dynamic exclusion was used with one repeat count, 15 s repeat duration and 60 s exclusion duration.

### 2.16. Database analysis and identification of modified residues

MS and MS/MS data were processed into Mascot generic format files and searched against the Swissprot database (version 201402) through the Mascot engine (v. 2.2) with the consideration of carbamidomethylation at cysteine, oxidation at methionine and N-hexosylation at Asparagine. The monoisotopic masses of 2 + or more charged peptides were searched with a peptide tolerance of 10 ppm and an MS/MS tolerance of 0.6 Da for fragment ions. Only peptides with a maximum of two missed cleavage sites were allowed in database searches. Positive identification of hexosylated peptides was performed by manual inspection of spectra. Peptides modified with extended glycan chains were investigated manually, and their corresponding MS/MS spectra were annotated. Here, XCalibur v. 2.2 sp1.48 was used for data processing, and MS deconvolution was performed by XtractRaw file from Thermo Scientific.

### 2.17. Construction of acceptor protein JC1

Amino acid residues 23–163 of Cj0114 from *C. jejuni* NCTC 11168 were used as a scaffold to design a novel acceptor protein. The native signal sequence from residues 1 to 23 was removed along with the native tetratricopeptide domain encoded within residues 164–315. Twelve NAT glycosylation sequons were added at the C-terminus of the new protein, each separated by a proline and a glycine. Finally, a hexa-histidine tag was added to the C-terminus to enable protein purification. This construct was DNA synthesized (Celtek Genes, USA) and subcloned into *Bam*HI and *Sph*I digested expression vector pACYC184.

## 3. Results

### 3.1. NGT and α6GlcT are required for adhesion of *Actinobacillus pleuropneumoniae* HS143 to A549 cell lines

Within the genome of *A. pleuropneumoniae* strain HS143, we identified two ORFs, orthologues of *apl_0104* (70% identity, BlastP) and *apl_0443* (82% identity, BlastP) in the L20 genome [25], coding for autotransporter adhesins*. In silico analysis* revealed that these adhesins have 75 and 95 N-X-(S/T) sequons, respectively (PROGLYCPROT). Naegeli *et al*. [17] carried out mass spectrometry analysis of *A. pleuropneumoniae*'s proteome for strain 4074 serotype 7, and the only glycopepetides identified belonged to two autotransporter adhesins [17], making these two adhesins the only native substrates for NGT identified so far. In NTHi*,* deletion of the *N*-linked glycosylation system results in a significantly reduced adherence phenotype [15]. In order to investigate whether this was the case for *A. pleuropneumoniae*, adhesion of WT HS143, isogenic mutants HS143Δ*ngt* and HS143Δ*agt* and its complements to A549 human adenocarcinoma lung epithelial cells was investigated.

**Figure 2.**
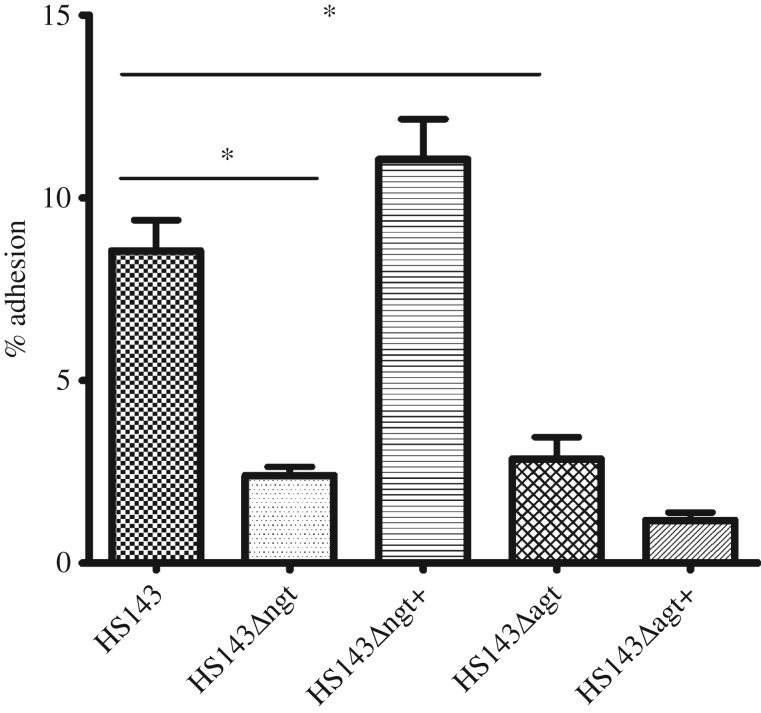
Percentage of adhesion of *A. pleuropneumoniae* strain HS143, isogenic mutants and complemented mutants to A549 cells infected at an MOI of 100 : 1 for 3 h prior to quantification of adherence. Horizontal bars indicate pairs of columns that are significantly different when compared with the wild-type HS143 (*p* < 0.05).

*Actinobacillus pleuropneumoniae* strain HS143, the wild-type strain, was found to have a percentage of adherent cells of 8.55 ± 0.84, *n* = 78 to A549 cells after 3 h incubation (figure 2). In order to understand the role of the cytoplasmic NGT in this adhesion phenotype, an in-frame deletion mutant of the *ngt* gene was generated in *A. pleuropneumoniae* and found to have a reduced percentage of adherent cells, 2.39 ± 0.25 (*n* = 78, *p* < 0.05), when compared with the wild-type. This phenotype was restored (11.05 ± 1.10, *n* = 30, *p* > 0.05) upon complementation with the *ngt* gene. Furthermore, when an α6GlcT deletion mutant in *A. pleuropneumoniae* was tested for adherence, it was observed that there was a decrease in adhesion (2.85 ± 0.60, *p* < 0.05, *n* = 30) to the same level as the NGT mutant. However, complementation with the ORF coding for α6GlcT was unable to rescue this phenotype (figure 2). Gentamycin treatment of cells confirmed that the bacterial counts observed were due to adhering and not invading bacteria.

### 3.2. *Agt* is part of a conserved putative operon that includes *ngt* and *rimO*

**Figure 3.**
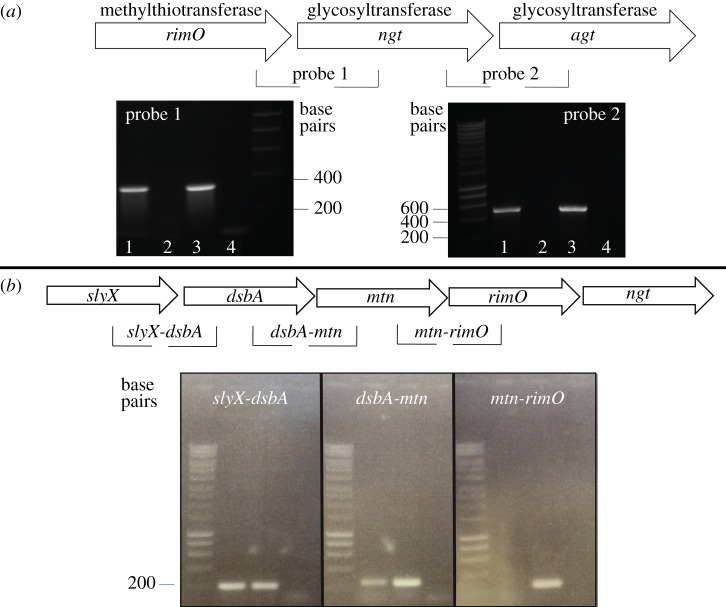
(*a*) Transcriptional analysis of the *A. pleuropneumoniae* NGT locus. Lane 1: cDNA as template; lane 2: RNA as template; lane 3: *A. pleuropneumoniae* HS143 genomic DNA positive control; lane 4: negative PCR control (no template). (*b*) Transcriptional analysis of the region upstream of *ngt*. Lane 1: cDNA as template; lane 2: genomic DNA positive control; lane 3: PCR control (RNA as template).

Unlike NTHi, adjacent to the *A. pleuropneumoniae N*-linking transferase gene, *ngt,* the flanking genes do not encode an adhesin or a dedicated adhesin transporter (figure 1). Instead*,* we identified an ORF coding for a protein with amino acid similarity to 30S ribosomal protein S12 methylthiotransferase, *rimO,* upstream of *ngt,* and a second glycosyltransferase-encoding gene downstream of *ngt*. Analysis of all available *A. pleuropneumoniae* genomes demonstrated that the genetic arrangement of the locus was absolutely conserved in all published *A. pleuropneumoniae* genomes and over 180 sequenced isolates (J.T.B. 2016, personal communication). Reverse transcriptase-PCR (RT-PCR) was used to analyse the expression of this locus in serovar 15 *A. pleuropneumoniae* reference strain HS143. Primers were designed spanning intergenic regions between the three ORFs. Probe 1 tested if an mRNA transcript was generated between *rimO* and *ngt*, and probe 2 tested for the presence of an mRNA transcript between *ngt* and *agt*. The results suggest that all three genes form an operon (figure 3*a*). Further RT-PCR analysis showed that the promoter driving *rimO* expression was independent of the ORF immediately upstream (figure 3*b*).

Messenger RNA was extracted at different time points during the growth of *A. pleuropneumoniae* and cDNA was generated by RT-PCR using a probe designed within *ngt.* This showed that *ngt* was transcribed at all-time points tested (electronic supplementary material, figure S1).

### 3.3. Absolute quantification of *rimO*, *ngt* and *agt* by qPCR

**Figure 4.**
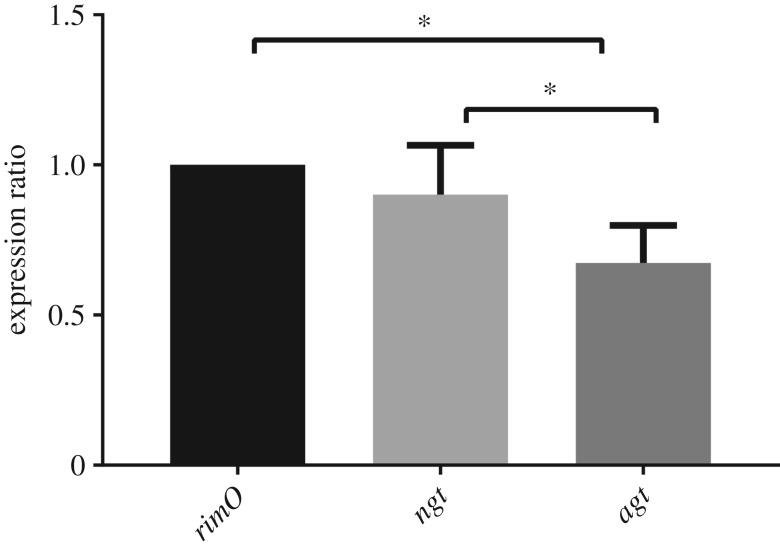
Absolute quantification of *rimO*, *ngt* and *agt* in *A. pleuropneumoniae* strain HS143 expressed in fold change. Horizontal bars indicate pairs of columns that are significantly different when compared with each other. Asterisk indicates *p* < 0.05 (*n* = 12).

In order to further validate the hypothesis that *rimO*, *ngt* and *agt* are co-transcribed, an absolute quantification qPCR was performed. The results were normalized by the absolute number of copies of *rimO* within each sample assuming *rimO* is the first ORF in the operon and therefore the closest to the putative promoter identified by bioinformatics analysis. A trend was observed in all four biological replicates (*n* = 12; 4 biologicals, 3 technical replicates) indicating a decrease in expression level from *rimO* to *agt* consistent with the genetic organization of the putative operon (*rimO* versus *ngt*, *p* < 0.05; *rimO* versus *agt*, *p* < 0.001; figure 4).

### 3.4. Reconstruction of the NGT glycosylation operon and its functional transfer and expression in *Escherichia coli*

**Figure 5.**
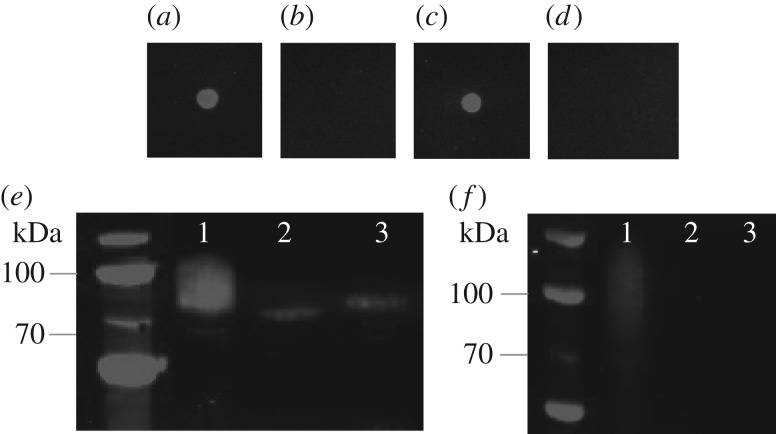
Glycosylation analysis of AtaC_1866–2428_ alongside NGT and α6GlcT by anti-dextran and anti-HIS western blots. Top panel: anti-dextran dot blot; (*a*) dextran; (*b*) AtaC with NGT K441A and functional α6GlcT; (*c*) AtaC with functional NGT and α6GlcT; (*d*) AtaC with functional NGT only. Bottom panel: anti-HIS and anti-dextran Western blots (*e,f*, respectively); lane 1, AtaC with functional NGT and α6GlcT; lane 2, AtaC with NGT K441A and functional α6GlcT; lane 3, AtaC with functional NGT only.

Following on from the RT-PCR studies indicating that both *agt* and *ngt* were co-transcribed, we amplified by PCR the two ORFs as a single amplicon and cloned them into the IPTG-inducible expression vector pEXT20 [33], to generate the plasmid pJC78. When the ORFs encoding α6GlcT and NGT were co-expressed with a fragment of an autotransporter adhesin from *A. pleuropneumoniae* (AtaC), which is a natural acceptor [17], a reduction in protein migration on SDS–PAGE was observed, indicating an increase in molecular weight consistent with the addition of an oligosaccharide (figure 5).

To further understand the *in vivo* glycosylation operon, individual mutations in *ngt* or *agt* were constructed within the plasmid pJC78. NGT activity was abolished by substituting the conserved lysine residue at position 441 by alanine (K441A) [18,20], whereas α6GlcT activity was abolished by the replacement of the leucine codon at amino acid position 7 with a stop codon (L7*). Schwarz *et al.* [19] indicated that *in vitro,* NGT and α6GlcT could assemble a glucose polymer between two and six residues on an acceptor peptide. We reasoned therefore that a commercially available antibody specific for α1–6 linked glucose tetrasaccharide (isomaltotetraose) may be able to detect and verify the nature of the polysaccharide generated by the cloned *agt–ngt* operon and of the knockouts. Ni–NTA purified proteins from the three construct combinations were tested for expression by dot blot analysis using an anti-dextran monoclonal antibody (mAb). This showed that a recognizable epitope could only be generated when NGT and α6GlcT were both functional (figure 5, top panel). SDS–PAGE and western blot analysis using an anti-HIS monoclonal antibody showed a smear visible above the point at which AtaC should migrate, but only when NGT and α6GlcT are both functional. This smearing was also detected using the anti-dextran monoclonal antibody (figure 5*f*, lane 1). AtaC glycosylated appears to migrate less than when detected by anti-HIS antibody, because the anti-dextran antibody will only recognize AtaC modified with four or more glucoses per site. Removing the function of NGT yielded an AtaC fragment that migrated to its unglycosylated location losing the epitope recognized by the anti-dextran mAb (figure 5*e*, lane 2). Finally, knocking out the function of α6GlcT reduced protein migration to a slightly higher level than that observed with NGT mutation alone (figure 5*e*, lane 3). This can be explained by glycosylation with a single hexose at multiple sites within the acceptor protein. Furthermore, this material was not recognized by anti-dextran mAb, suggesting that glycosylation had occurred, but that no polymer had been generated (figure 5*f*, lane 3).

### 3.5. Confirmation of hexose build-up on AtaC

**Figure 6:**
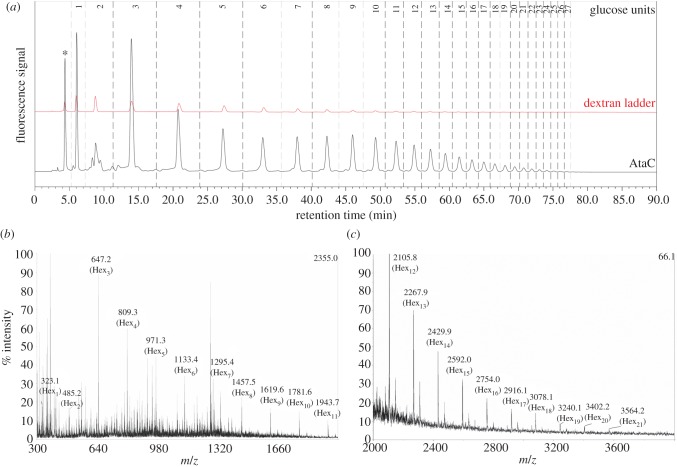
(*a*) NP-HPLC analysis of 2-AB labelled glycans released from purified AtaC which was co-expressed with NGT and α6GlcT in *E. coli* DH10β (black). A glucose homopolymer (dextran) ladder serves as a reference for retention time (red). The peak originating from excess 2-AB label (rt = 4.1 min) is marked with an asterisk. (*b,c*) MALDI mass spectrometry analysis of the same glycan sample confirms the identity of the observed glycan chains as a hexose polymer.

In order to confirm the identity of the observed post-translational modification of AtaC, the glycans from purified protein were released by hydrazinolysis, fluorescently labelled with 2-aminobenzamide (2-AB) and analysed by normal-phase HPLC (figure 6*a*).

With a labelled dextran ladder used as a reference, this analysis revealed the presence of glycan chains of varying lengths (1–27 monosaccharide units). This was further confirmed by MALDI-MS analysis. Peaks differing in mass by 162 Da suggested potential for glucose or galactose attachment (figure 6*b,c*). Therefore, the results from both methods were in agreement showing a hexose polymer ranging up to at least 20 units.

**Figure 7.**
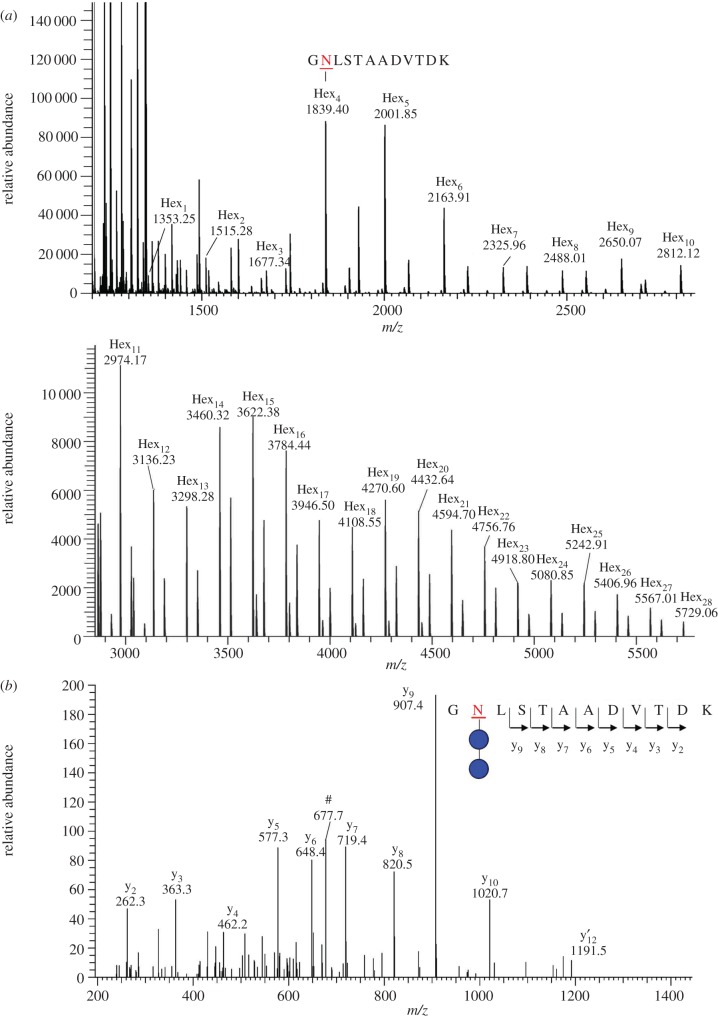
Deconvoluted MS spectra show that peptide GNLSTAADVTDK from AtaC is modified with a hexose polymer ranging from 1 to 28 units. (*b*) MS/MS spectrum of *m*/*z* 758.3497(+2), corresponding to GNLSTAADVTDK modified with two hexoses, showed continuous fragmentation ions, which confirm the peptide identity. The hash tag marks doubly charged ion with neutral loss of hexose from precursor ion. y’ indicates the y ion without hexoses.

To confirm that particular sites on the protein were modified with these elongated glycans, LC–ESI–MS/MS analysis of glycosylated AtaC was performed (figure 7 and table 1). This showed that previously identified glycosylation sites were occupied [17]. In total, 15 asparagine (Asn) residues were identified as being modified with glycan chains of variable length. For example, glycopeptide GNLSTAADVTDK could be detected modified with an *N*-linked glycan consisting of 1–29 hexose units (table 1). On other sites, only short glycan chains could be detected, whereas one peptide (NISTVVK) could only be detected as being modified with glycan chains of more than 14 hexoses. These results are summarized in table 1 and confirm western blot evidence that co-expression of NGT and α6GlcT leads to the formation of Asn-linked, linear hexose chains of up to 29 units in length.

**Table 1.** Summary of glycosylation status for each site from AtaC. Underlined letters: in red, N-X-S/T sequons; in blue, asparagine residues found to be occupied but not part of an N-X-S/T consensus sequon.

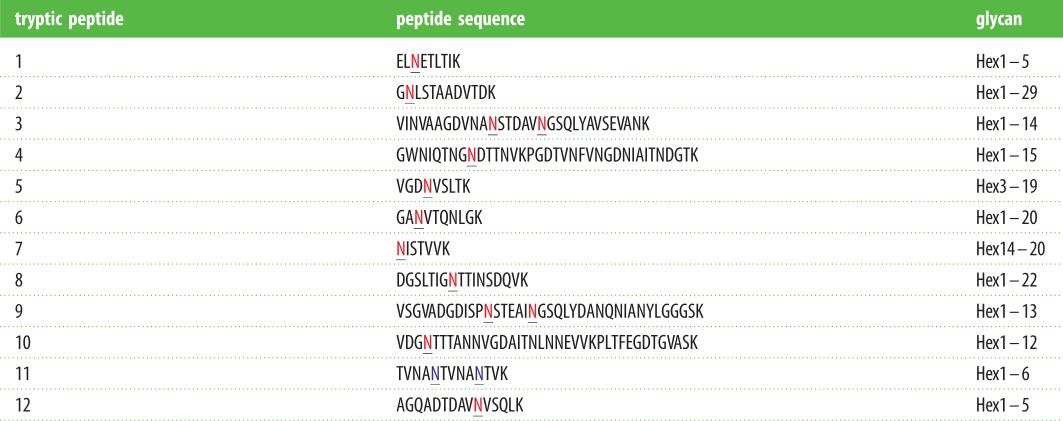

### 3.6. The *ngt/agt* operon can be used to modify alternative substrates with dextran

**Figure 8.**
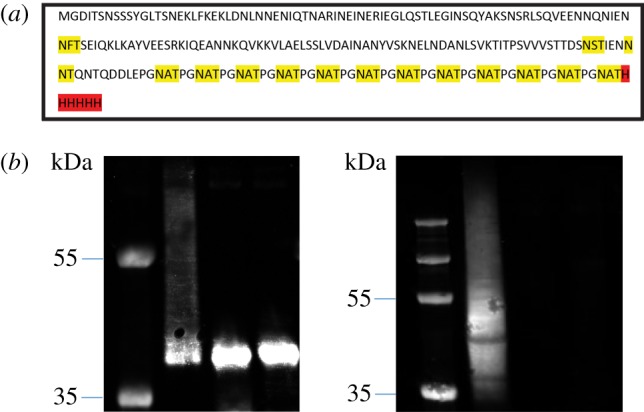
Glycosylation of engineered acceptor protein (JC1) by NGT and α6GlcT. (*a*) Amino acid sequence of the new target glycoprotein JC1. Highlighted in yellow are glycosylation sequons, and in red, the hexa-HIS tag used for protein purification. (*b*) Glycosylation of the acceptor protein JC1 with NGT and α6GlcT. Left panel: anti-histidine tag western blot; right panel, anti-dextran western blot. Lane 1: JC1 expressed with functional NGT and α6GlcT; lane 2: JC1 with NGT K441A and α6GlcT; lane 3: JC1 with NGT but non-functional α6GlcT*.*

Following assembly of plasmid pJC78, we began testing if the *ngt/agt* operon could be used to make *N*-linked glucose polymers on non-native substrate proteins in a similar manner to NGT alone [17]. We selected Cj0114 from the ɛ-proteobacterium *Campylobacter jejuni* as a scaffold for designing a new acceptor protein. The native Cj0114 tetratricopeptide domain was removed to reduce protein toxicity and simplify purification. At the *C*-terminus of the protein, the 12 added NAT glycosylation sequons were followed by a hexa-histidine tag to enable protein purification. The new protein, named JC1, was constitutively expressed from the plasmid pJC1. Combining the plasmids pJC1 and pJC78 generated an epitope that could be recognized by the anti-dextran mouse mAb; this disappeared upon knocking out the function of *ngt* or a*gt* (figure 8). The marginally different sizes in the anti-His and anti-dextran western blot are due to the recognition epitope for the anti-dextran antibody, where only highly polymerized proteins are detected (acceptors modified with four or more glucose residues). These findings indicated that NGT and α6GlcT can be made to target any protein.

## 4. Discussion

Novel bacterial glycosylation systems are regularly being discovered as glycan analyses methodologies improve [34–37]. The functions of these glycosylation systems are yet to be fully appreciated, but it is now apparent that glycosylation is a feature common to most bacteria.

In this study, we report the investigation of a cytoplasmic glycosylation system in a member of the *Pasteurellaceae* family, *A. pleuropneumoniae*. Our results demonstrate that despite similarities between NGT and its orthologue, HMW1C, in NTHi, the system described here is unique. The *A. pleuropneumoniae N*-linking locus consists of two co-transcribed glycosyltransferases (*ngt* and *agt*) with no associated adhesin or transporter. Another significant difference between the *A. pleuropneumoniae* system and that of NTHi is that the promoter for *rimO*, upstream of *ngt*, appears to be responsible for driving transcription of *ngt* and *agt* (figure 3). Recent studies have shown that in *Aggregibacter aphrophilus* and *Haemophilus ducreyi* [16,21] *hmwC* is also located downstream of *rimO*, although a transcriptional link has yet to be proven [21]. In *E. coli*, RimO is an enzyme that catalyses the methylthiolation of ribosomal subunit S12 at the universally conserved D88 residue. Furthermore, it has been shown that knocking out *rimO* in *E. coli* leads to a growth defect [38,39]. The significance of RimO has also been reported in *Thermus thermophilus*, where residue D88 cannot be mutated [40], leading to the conclusion that although methylthiolation is not essential in every organism, RimO clearly plays an important role in maintaining bacterial fitness [39]. Our tests indicate that the *A. pleuropneumoniae rimO* promoter is active at every time point tested, suggesting that *ngt* and *agt* are constitutively expressed (electronic supplementary material, figure S1)*,* and therefore the cytoplasmic *N*-linking glycosylation system is always available to modify substrate proteins. Furthermore, qPCR analysis of the locus indicated transcriptional levels consistent with an operonic structure, where the highest level of transcription detected was of *rimO*, followed by *ngt* and *agt*, respectively (figure 4). This is in agreement with the findings reported by Lim *et al*. [41] whereby the expression level of the genes proximal to the promoter was greater than the ones farthest from it. Bioinformatic analysis of the DNA sequence surrounding the putative *rimO/ngt/agt* operon identifies a transcriptional promoter just upstream of *rimO* and a Rho-independent terminator downstream of *agt* (electronic supplementary material, figure S2) [42]. Nevertheless, to gain further insights into the regulation of the locus, other approaches such as RNAseq could be carried out. Furthermore, *in silico* analysis of all publically available genomes and over 180 others (J.T.B. 2016, personal communication) indicates that the gene order is absolutely conserved (data not shown).

In this work, we also demonstrate that NGT plays an important biological role in the ability of *A. pleuropneumoniae* to adhere to A549 human adenocarcinoma lung epithelial cells, which, although from human origin, are from biologically relevant tissue. The rationale for using A549 cells, instead of St Jude Porcine lung (SJPL) cells, which have been widely used to assess *A. pleuropneumoniae* adhesion [4,43,44], was that the SJPL cell line was found to be misclassified, and is simian in origin [45]. In order to draw absolute conclusions regarding the role of this N-linked glycosylation system in aiding *A. pleuropneumoniae* pathogenesis in the pig, the adhesion assay data that obtained in this study could be extended to investigate other tissues such as *ex vivo* organ cultures [46] possibly primary cell cultures from pig lung epithelial cells.

Similarly to our study, a significant reduction in adherence was reported for *E. coli* expressing the cloned hmw1 locus from NTHi when the function of HMW1C (the NGT orthologue) was removed [15]. The *hmw1*/*hmw2* loci and the *ngt* operon differ in that the NTHi loci encode an adhesin and an adhesin transporter alongside *hmwC*, whereas the *A. pleuropneumoniae* locus encodes an α6GlcT polymerizing glucosyltransferase (figure 1). Surprisingly, knocking out the function of the α6GlcT transferase also resulted in a significant reduction in adherence comparable to that detected when NGT activity was abolished. Plasmid-based complementation with *agt* only (figure 2) and *ngt/agt* (data not shown) proved insufficient to rescue the adhesion phenotype seen with the wild-type. Our failed attempts to rescue the phenotype suggest a need for fine transcriptional control of *agt* levels in the bacterium. It is possible that, when *ngt* is being expressed in the chromosome and *agt* is on a plasmid, over-glycosylation of target sites occurs, resulting in incorrect adhesin structural conformation. Potentially, this incorrect folding could prevent surface presentation or adhesin function.

Some of the best-studied examples of glycosylated autotransporter adhesins are *O*-linked, and found in *E. coli. O*-linked glycosyltransferases can glycosylate TibA, Ag43 and AIDA-I in their passenger domains [47–49]. Bioinformatic analysis of the autotransporter adhesin used in our study, AtaC from *A. pleuropneumoniae* AP76 (GenBank accession number ACE61172.1), indicated the presence of 72 NX-(S/T) sequons. The majority of these (59/72) are localized in the passenger domain of the adhesin, further demonstrating the similarities with the *O*-linked counterparts. Whether glycosylation is required, so that the adhesin assumes the correct conformation and is not degraded as observed in AIDA-I, or if it is so that the adhesin can adopt a conformation suited for adhesion as described in TibA [50], remains to be determined even in these well-studied proteins.

Our demonstration of glucose polymer assembly within *E. coli* cells, when the *ngt/agt* locus is overexpressed alongside an acceptor protein (figure 5), led us to investigate if this glycan could be detected on the surface of *A. pleuropneumoniae*. Immunofluorescence studies using an anti-dextran antibody that recognizes isomaltotetraose as a minimum epitope failed to detect any signal, even in permeabilized cells (data not shown). This suggests that although the capability exists to form α-1,6 glucose chains greater than four subunits heterologously, in *A. pleuropneumoniae* the necessary epitope for detection with anti-dextran antibody does not appear to be formed. Analysis of the glycosylated peptides generated within *E. coli*, as determined by peak quantification of the HPLC chromatogram (figure 6), revealed a steady decrease in abundance of the oligosaccharide with increasing chain length. It was however noteworthy that the peak corresponding to Glc_2_-2AB (retention time: 8.8 min) was considerably smaller than the peaks corresponding to Glc_1_-2AB and Glc_3_-2AB, indicating that the addition of the first α1–6-linked glucose might be considerably slower than the subsequent transfer reactions. This suggests that the first α6GlcT-catalysed reaction is, in fact, the rate-limiting step in the biosynthesis of these extended *N*-glycan chains.

In a review of the HMWC literature, we found instances where adhesin glycopeptides with dihexose modifications have been reported, in the absence of a co-localized ORF-like *agt* [15,51]. Rempe *et al*. [21] report dihexose modifications on four glycopeptides belonging to the autotransporter adhesin of *K. kingae*. These raise several possibilities; the first is that the reported glycopeptides actually contain two individual hexose attachments and not two hexoses together. Second, it may be possible that the HmwC from *K. kingae* is able to catalyse *N*-linked attachment and subsequent polymerization. Third, one cannot rule out there may be another glycosyltransferase in the genome that is enabling dihexose assembly.

A review of the NGT-specific literature reveals an interesting disparity in the function of this enzyme when tested *in vitro* and *in vivo.* Choi *et al*. [51] reported that *in vitro,* NGT is capable of forming dihexoses. However, this study indicates that α6GlcT is essential for glycosidic bond formation and extension of the glucose polymer *in vivo*. This is in agreement with a previous study by Naegeli *et al.*, which failed to detect any polymerization when NGT alone was expressed in *E. coli* to glycosylate an acceptor protein [17].

Our finding that α6GlcT function is necessary to maintain adhesion in *A. pleuropneumoniae* indicates that this enzyme must be extending glucose residues at some sites within the autotransporter adhesins. However, by transferring the *N*-linking glycosylation locus into *E. coli*, we showed that α6GlcT and NGT are unable to fully complement each other's functions. Our study also provides further evidence that ‘orphan’ HMWC family of enzymes that have not evolved to be co-localized with their target substrate continue to modify proteins involved in adhesion. It is noteworthy that every bacterial species reported thus far with this genetic arrangement uses the glycosylation system to target autotransporter adhesins [16,21]. Glycosylation has been linked to protection from proteolytic degradation, correct protein folding and correct transport to the surface, all of which would have an effect on cell adhesion. Further studies are ongoing to ascertain the level of interaction between α6GlcT/NGT and the target protein(s).

By demonstrating how to harness the *ngt/agt* operon, we have shown potential for glycoengineering applications, including the generation of *N*-linked glucose-based conjugate vaccines against *A. pleuropneumoniae*. The genetic conservation of the *ngt* operon in *A. pleuropneumoniae* would favour the development of a glycoconjugate vaccine against multiple *A. pleuropneumoniae* serovars. Other potential applications include the development of dextran-based conjugates that may be useful against bacteria such as *Helicobacter pylori* [52]*.* Recently, such conjugates have been shown to be immunogenic, and post-immune sera from rabbits vaccinated with dextran-based conjugates exhibited activity against strains of *H. pylori* that contain α(1–6) glucose as part of their LPS [52].

The field of bacterial glycobiology is burgeoning and investigations into various glycosylation systems, such as the NGT/α6GlcT system reported here, help to understand their functional roles. Our results demonstrate the importance of genetic and phenotypic screens for investigating glycosylation systems, and that this data can directly benefit bacterial glycoengineering.

## Supplementary Material

Glycosylation system investigation, Terra et al. Table S1 ;Glycosylation system investigation, Terra et al. Table S2;Glycosylation system investigation, Terra et al. Figure S1;Glycosylation system investigation, Terra et al. Figure S2

## Data accessibility

The datasets supporting this article have been uploaded as part of the electronic supplementary material.

## References

[1] ProHealth 2015 Production diseases: the cost to pig producers. See www.fp7-prohealth.eu/news-index/newsletter-november-2015/production-diseases-cost-pig-producers.

[2] Dom P, Haesebrouck F, Ducatelle R, Charlier G 1994 *In vivo* association of *Actinobacillus pleuropneumoniae* serotype 2 with the respiratory epithelium of pigs. Infect. Immun. 62, 1262–1267. 7907578 10.1128/iai.62.4.1262-1267.1994 PMC186267

[3] Boekema BK, Stockhofe-Zurwieden N, Smith HE, Kamp EM, van Putten JP, Verheijden JH 2003 Adherence of *Actinobacillus pleuropneumoniae* to primary cultures of porcine lung epithelial cells. Vet. Microbiol. 93, 133–144. (doi:10.1016/S0378-1135(03)00020-8) 12637001 10.1016/s0378-1135(03)00020-8

[4] Auger E, Deslandes V, Ramjeet M, Contreras I, Nash JHE, Harel J, Gottschalk M, Olivier M, Jacques M 2009 Host-pathogen interactions of *Actinobacillus pleuropneumoniae* with porcine lung and tracheal epithelial cells. Infect. Immun. 77, 1426–1441. (doi:10.1128/IAI.00297-08) 19139196 10.1128/IAI.00297-08 PMC2663157

[5] Sarkozi R, Makrai L, Fodor L 2015 Identification of a proposed new serovar of *Actinobacillus Pleuropneumoniae*: Serovar 16. Acta Vet. Hung. 63, 444–450. (doi:10.1556/004.2015.041) 26599091 10.1556/004.2015.041

[6] Van Overbeke I, Chiers K, Charlier G, Vandenberghe I, Van Beeumen J, Ducatelle R, Haesebrouck F 2002 Characterization of the *in vitro* adhesion of *Actinobacillus pleuropneumoniae* to swine alveolar epithelial cells. Vet. Microbiol. 88, 59–74. (doi:10.1016/S0378-1135(02)00080-9) 12119138 10.1016/s0378-1135(02)00080-9

[7] Ramjeet M, Deslandes V, St Michael F, Cox AD, Kobisch M, Gottschalk M, Jacques M 2005 Truncation of the lipopolysaccharide outer core affects susceptibility to antimicrobial peptides and virulence of *Actinobacillus pleuropneumoniae* serotype 1. J. Biol. Chem. 280, 39 104–39 114. (doi:10.1074/jbc.M502852200) 10.1074/jbc.M502852200 16188878

[8] Howard SL et al. 2009 *Campylobacter jejuni* glycosylation island important in cell charge, legionaminic acid biosynthesis, and colonization of chickens. Infect. Immun. 77, 2544–2556. (doi:10.1128/IAI.01425-08) 19307210 10.1128/IAI.01425-08 PMC2687346

[9] Iwashkiw JA et al. 2012 Identification of a general O-linked protein glycosylation system in *Acinetobacter baumannii* and its role in virulence and biofilm formation. PLoS Pathog. 8, e1002758 (doi:10.1371/journal.ppat.1002758) 22685409 10.1371/journal.ppat.1002758 PMC3369928

[10] Ciocchini AE, Rey Serantes DA, Melli LJ, Iwashkiw JA, Deodato B, Wallach J, Feldman MF, Ugalde JE, Comerci DJ 2013 Development and validation of a novel diagnostic test for human brucellosis using a glyco-engineered antigen coupled to magnetic beads. PLoS Negl. Trop. Dis. 7, e2048 (doi:10.1371/journal.pntd.0002048) 23459192 10.1371/journal.pntd.0002048 PMC3573069

[11] Wacker M et al. 2014 Prevention of *Staphylococcus aureus* infections by glycoprotein vaccines synthesized in *Escherichia coli*. J. Infect. Dis. 209, 1551–1561. (doi:10.1093/infdis/jit800) 24308931 10.1093/infdis/jit800 PMC3997581

[12] Wacker M et al. 2002 N-linked glycosylation in *Campylobacter jejuni* and its functional transfer into *E. coli*. Science 298, 1790–1793. (doi:10.1126/science.298.5599.1790) 12459590 10.1126/science.298.5599.1790

[13] Linton D et al. 2005 Functional analysis of the *Campylobacter jejuni* N-linked protein glycosylation pathway. Mol. Microbiol. 55, 1695–1703. (doi:10.1111/j.1365-2958.2005.04519.x) 15752194 10.1111/j.1365-2958.2005.04519.x

[14] Twine SM, Reid CW, Aubry A, McMullin DR, Fulton KM, Austin J, Logan SM 2009 Motility and flagellar glycosylation in *Clostridium difficile*. J. Bacteriol. 191, 7050–7062. (doi:10.1128/JB.00861-09) 19749038 10.1128/JB.00861-09 PMC2772495

[15] Grass S, Buscher AZ, Swords WE, Apicella MA, Barenkamp SJ, Ozchlewski N, St Geme JW 2003 The *Haemophilus influenzae* HMW1 adhesin is glycosylated in a process that requires HMW1C and phosphoglucomutase, an enzyme involved in lipooligosaccharide biosynthesis. Mol. Microbiol. 48, 737–751. (doi:10.1046/j.1365-2958.2003.03450.x) 12694618 10.1046/j.1365-2958.2003.03450.x

[16] McCann JR, St Geme JW III 2014 The HMW1C-like glycosyltransferases--an enzyme family with a sweet tooth for simple sugars. PLoS Pathog. 10, e1003977 (doi:10.1371/journal.ppat.1003977) 24722584 10.1371/journal.ppat.1003977 PMC3983070

[17] Naegeli A, Neupert C, Fan YY, Lin CW, Poljak K, Papini AM, Schwarz F, Aebi M 2014 Molecular analysis of an alternative N-glycosylation machinery by functional transfer from *Actinobacillus pleuropneumoniae* to *Escherichia col*i. J. Biol. Chem. 289, 2170–2179. (doi:10.1074/jbc.M113.524462) 24275653 10.1074/jbc.M113.524462 PMC3900963

[18] Naegeli A, Michaud G, Schubert M, Lin CW, Lizak C, Darbre T, Reymond J-L, Aebi M 2014 Substrate specificity of cytoplasmic N-glycosyltransferase. J. Biol. Chem. 289, 24 521–24 532. (doi:10.1074/jbc.M114.579326) 10.1074/jbc.M114.579326 PMC4148877 24962585

[19] Schwarz F, Fan YY, Schubert M, Aebi M 2011 Cytoplasmic N-glycosyltransferase of *Actinobacillus pleuropneumoniae* is an inverting enzyme and recognizes the NX(S/T) consensus sequence. J. Biol. Chem. 286, 35 267–35 274. (doi:10.1074/jbc.M111.277160) 21852240 10.1074/jbc.M111.277160 PMC3186387

[20] Kawai F, Grass S, Kim Y, Choi K-J, St Geme JW, Yeo H-J 2011 Structural insights into the glycosyltransferase activity of the *Actinobacillus pleuropneumoniae* HMW1C-like protein. J. Biol. Chem. 286, 38 546–38 557. (doi:10.1074/jbc.M111.237602) 10.1074/jbc.M111.237602 PMC3207471 21908603

[21] Rempe KA, Spruce LA, Porsch EA, Seeholzer SH, Nørskov-Lauritsen N, St Geme JW 2015 Unconventional N-Linked Glycosylation Promotes Trimeric Autotransporter Function in *Kingella kingae* and *Aggregatibacter aphrophilus*. MBio 6, e01206-15. (doi:10.1128/mBio.01206-15) 10.1128/mBio.01206-15 PMC4550697 26307167

[22] Xiao L et al. 2012 Apa is a trimeric autotransporter adhesin of *Actinobacillus pleuropneumoniae* responsible for autoagglutination and host cell adherence. J. Basic Microbiol. 52, 598–607. (doi:10.1002/jobm.201100365) 22143982 10.1002/jobm.201100365

[23] Ferrieres L, Hemery G, Nham T, Guerout AM, Mazel D, Beloin C, Ghigo J-M 2010 Silent mischief: bacteriophage Mu insertions contaminate products of *Escherichia coli* random mutagenesis performed using suicidal transposon delivery plasmids mobilized by broad-host-range RP4 conjugative machinery. J. Bacteriol. 192, 6418–6427. (doi:10.1128/JB.00621-10) 20935093 10.1128/JB.00621-10 PMC3008518

[24] Cuccui J, Easton A, Chu KK, Bancroft GJ, Oyston PCF, Titball RW, Wren BW 2007 Development of signature-tagged mutagenesis in *Burkholderia pseudomallei* to identify genes important in survival and pathogenesis. Infect. Immun. 75, 1186–1195. (doi:10.1128/IAI.01240-06) 17189432 10.1128/IAI.01240-06 PMC1828585

[25] Foote SJ, Bosse JT, Bouevitch AB, Langford PR, Young NM, Nash JH 2008 The complete genome sequence of *Actinobacillus pleuropneumoniae* L20 (serotype 5b). J. Bacteriol. 190, 1495–1496. (doi:10.1128/JB.01845-07) 18065534 10.1128/JB.01845-07 PMC2238223

[26] Bosse JT et al. 2014 The generation of successive unmarked mutations and chromosomal insertion of heterologous genes in *Actinobacillus pleuropneumoniae* using natural transformation. PLoS ONE 9, e111252 (doi:10.1371/journal.pone.0111252) 25409017 10.1371/journal.pone.0111252 PMC4237320

[27] Bosse JT, Nash JH, Kroll JS, Langford PR 2004 Harnessing natural transformation in *Actinobacillus pleuropneumoniae*: a simple method for allelic replacements. FEMS Microbiol. Lett. 233, 277–281. (doi:10.1111/j.1574-6968.2004.tb09492.x) 15063496 10.1016/j.femsle.2004.02.022

[28] Bosse JT, Durham AL, Rycroft AN, Kroll JS, Langford PR 2009 New plasmid tools for genetic analysis of *Actinobacillus pleuropneumoniae* and other pasteurellaceae. Appl. Environ. Microbiol. 75, 6124–6131. (doi:10.1128/AEM.00809-09) 19666733 10.1128/AEM.00809-09 PMC2753053

[29] Pumirat P, Cuccui J, Stabler RA, Stevens JM, Muangsombut V, Singsuksawat E, Stevens MP, Wren BW, Korbsrisate S 2010 Global transcriptional profiling of *Burkholderia pseudomallei* under salt stress reveals differential effects on the Bsa type III secretion system. BMC Microbiol. 10, 171 (doi:10.1186/1471-2180-10-171) 20540813 10.1186/1471-2180-10-171 PMC2896371

[30] Nielsen KK, Boye M 2005 Real-time quantitative reverse transcription-PCR analysis of expression stability of *Actinobacillus pleuropneumoniae* housekeeping genes during *in vitro* growth under iron-depleted conditions. Appl. Environ. Microbiol. 71, 2949–2954. (doi:10.1128/AEM.71.6.2949-2954.2005) 15932989 10.1128/AEM.71.6.2949-2954.2005 PMC1151834

[31] Bigge JC, Patel TP, Bruce JA, Goulding PN, Charles SM, Parekh RB 1995 Nonselective and efficient fluorescent labeling of glycans using 2-amino benzamide and anthranilic acid. Anal. Biochem. 230, 229–238. (doi:10.1006/abio.1995.1468) 7503412 10.1006/abio.1995.1468

[32] Wisniewski JR, Zougman A, Nagaraj N, Mann M 2009 Universal sample preparation method for proteome analysis. Nat. Methods. 6, 359–362. (doi:10.1038/nmeth.1322) 19377485 10.1038/nmeth.1322

[33] Dykxhoorn DM, St Pierre R, Linn T 1996 A set of compatible tac promoter expression vectors. Gene 177, 133–136. (doi:10.1016/0378-1119(96)00289-2) 8921858 10.1016/0378-1119(96)00289-2

[34] Young NM et al. 2002 Structure of the N-linked glycan present on multiple glycoproteins in the Gram-negative bacterium, *Campylobacter jejuni*. J. Biol. Chem. 277, 42 530–42 539. (doi:10.1074/jbc.M206114200) 10.1074/jbc.M206114200 12186869

[35] Pustylnikov S, Sagar D, Jain P, Khan ZK 2014 Targeting the C-type lectins-mediated host-pathogen interactions with dextran. J. Pharm. Pharm. Sci. 17, 371–392. (doi:10.18433/J3N590) 25224349 10.18433/j3n590 PMC5553543

[36] Cuskin F et al. 2015 Human gut Bacteroidetes can utilize yeast mannan through a selfish mechanism. Nature 517, 165–169. (doi:10.1038/nature13995) 25567280 10.1038/nature13995 PMC4978465

[37] Harding CM et al. 2015 Acinetobacter strains carry two functional oligosaccharyltransferases, one devoted exclusively to type IV pilin, and the other one dedicated to O-glycosylation of multiple proteins. Mol. Microbiol. 96, 1023–1041. (doi:10.1111/mmi.12986) 25727908 10.1111/mmi.12986

[38] Kowalak JA, Walsh KA 1996 Beta-methylthio-aspartic acid: identification of a novel posttranslational modification in ribosomal protein S12 from *Escherichia coli*. Protein Sci. 5, 1625–1632. (doi:10.1002/pro.5560050816) 8844851 10.1002/pro.5560050816 PMC2143476

[39] Anton BP, Saleh L, Benner JS, Raleigh EA, Kasif S, Roberts RJ 2008 RimO, a MiaB-like enzyme, methylthiolates the universally conserved Asp88 residue of ribosomal protein S12 in *Escherichia coli*. Proc. Natl Acad. Sci. USA 105, 1826–1831. (doi:10.1073/pnas.0708608105) 18252828 10.1073/pnas.0708608105 PMC2538847

[40] Carr JF, Hamburg D-M, Gregory ST, Limbach PA, Dahlberg AE 2006 Effects of streptomycin resistance mutations on posttranslational modification of ribosomal protein S12. J. Bacteriol. 188, 2020–2023. (doi:10.1128/JB.188.5.2020-2023.2006) 16484214 10.1128/JB.188.5.2020-2023.2006 PMC1426572

[41] Lim HN, Lee Y, Hussein R 2011 Fundamental relationship between operon organization and gene expression. Proc Natl Acad. Sci. USA 108, 10 626–10 631. (doi:10.1073/pnas.1105692108) 10.1073/pnas.1105692108 PMC3127940 21670266

[42] Solovyev AS 2011 Automatic annotation of microbial genomes and metagenomic sequences. In Metagenomics and its applications in agriculture, biomedicine and environmental studies (ed. Li RW), pp. 61–78. Hauppauge, NY: Nova Science Publishers.

[43] Levesque C, Provost C, Labrie J, Hernandez Reyes Y, Burciaga Nava JA, Gagnon CA, Jacques M 2014 Actinobacillus pleuropneumoniae possesses an antiviral activity against porcine reproductive and respiratory syndrome virus. PLoS ONE 9, e98434 (doi:10.1371/journal.pone.0098434) 24878741 10.1371/journal.pone.0098434 PMC4039538

[44] Liu J, Hu L, Xu Z, Tan C, Yuan F, Fu S, Cheng H, Chen H, Bei W 2015 *Actinobacillus pleuropneumoniae* two-component system QseB/QseC regulates the transcription of PilM, an important determinant of bacterial adherence and virulence. Vet. Microbiol. 177, 184–192. (doi:10.1016/j.vetmic.2015.02.033) 25796134 10.1016/j.vetmic.2015.02.033

[45] Silversides DW, Music N, Jacques M, Gagnon CA, Webby R 2010 Investigation of the species origin of the St. Jude Porcine Lung epithelial cell line (SJPL) made to researchers. J. Virol. 84, 5454–5455. (doi:10.1128/JVI.00042-10) 20200241 10.1128/JVI.00042-10 PMC2863845

[46] Dugal F, Belanger M, Jacques M 1992 Enhanced adherence of *Pasteurella multocida* to porcine tracheal rings preinfected with *Bordetella bronchiseptica*. Can. J. Vet. Res. 56, 260–264. 1423064 PMC1263549

[47] Elsinghorst EA, Weitz JA 1994 Epithelial cell invasion and adherence directed by the enterotoxigenic *Escherichia coli* tib locus is associated with a 104-kilodalton outer membrane protein. Infect. Immun. 62, 3463–3471. 8039917 10.1128/iai.62.8.3463-3471.1994 PMC302979

[48] Benz I, Schmidt MA 2001 Glycosylation with heptose residues mediated by the aah gene product is essential for adherence of the AIDA-I adhesin. Mol. Microbiol. 40, 1403–1413. (doi:10.1046/j.1365-2958.2001.02487.x) 11442838 10.1046/j.1365-2958.2001.02487.x

[49] van der Woude MW, Henderson IR 2008 Regulation and function of Ag43 (flu). Annu. Rev. Microbiol. 62, 153–169. (doi:10.1146/annurev.micro.62.081307.162938) 18785838 10.1146/annurev.micro.62.081307.162938

[50] Cote J-P, Charbonneau M-E, Mourez M 2013 Glycosylation of the *Escherichia coli* TibA self-associating autotransporter influences the conformation and the functionality of the protein. PLoS ONE 8, e80739 (doi:10.1371/journal.pone.0080739) 24278316 10.1371/journal.pone.0080739 PMC3835316

[51] Choi K-J, Grass S, Paek S, St Geme JW 3rd, Yeo H-J 2010 The *Actinobacillus pleuropneumoniae* HMW1C-like glycosyltransferase mediates N-linked glycosylation of the *Haemophilus influenzae* HMW1 adhesin. PLoS ONE 5, e15888 (doi:10.1371/journal.pone.0015888) 21209858 10.1371/journal.pone.0015888 PMC3012730

[52] Altman E, Chandan V, Harrison B 2014 The potential of dextran-based glycoconjugates for development of *Helicobacter pylori* vaccine. Glycoconj. J. 31, 13–24. (doi:10.1007/s10719-013-9496-4) 23990317 10.1007/s10719-013-9496-4

