# Supplementary material for: The N-linking glycosylation system from Actinobacillus pleuropneumoniae is required for adhesion and has potential use in glycoengineering
Source: Open Biol. 2017 Jan 11;7(1):160212. doi: 10.1098/rsob.160212 (PMC5303269; doi:10.1098/rsob.160212)
Supplement: Glycosylation system investigation, Terra et al. Table S1 ;Glycosylation system investigation, Terra et al. Table S2;Glycosylation system investigation, Terra et al. Figure S1;Glycosylation system investigation, Terra et al. Figure S2 [file rsob160212supp1.docx]

**The *N*-linking glycosylation system from *Actinobacillus pleuropneumoniae* is required for adhesion and has potential use in glycoengineering**

Jon Cuccui^1^*, Vanessa S. Terra^1^*, Janine T. Bossé^2^, Andreas Naegeli^3,4^, Sherif Abouelhadid^1^, Yanwen Li^2^, Chia-Wei Lin^3^, Prerna Vohra^1^, Alexander W. Tucker^4^, Andrew N. Rycroft^5^, Duncan J. Maskell^4^, Markus Aebi^3^, Paul R. Langford^2^, Brendan W. Wren^1^**, on behalf of the BRaDP1T consortium

**Supplementary figures and tables**

**Table S1** Strains and plasmids used in this study.

| **Sample** | **Description** | **Source/Reference** |
| --- | --- | --- |
| *A. pleuropneumoniae* serovar 15 strain | Strain HS143, NAD-dependent, V factor dependent*, apxIBD,apxIICA,apxIIICA ApxIIIBD apxIVA* positive by PCR | (47) |
| *A. pleuropneumoniae*  serovar 15 Δ*ngt* | *A. pleuropneumoniae* NGT knockout | This study |
| *A. pleuropneumoniae*  serovar 15 Δ*agt* | *A. pleuropneumoniae* α6GlcT knockout | This study |
| *E. coli* MFDpir | MG1655 RP4-2-Tc::[*Mu1::aac(3)IV-ΔaphA-Δnic35-_Mu2::zeo*] *dap*A::(erm-pir) Δ*recA* | (17) |
| *E. coli* TOP10 | *F- mcrA Δ(mrr-hsdRMS-mcrBC) φ80lacZΔM15 ΔlacX74 nupG recA1 araD139 Δ(ara-leu)7697 galE15 galK16 rpsL(Str^R^) endA1 λ*^-^ | Invitrogen |
| *E. coli* DH10β | *F´ proA+B+ lacI^q^ ∆ lacZ M15/ fhuA2 ∆(lac-proAB) glnV gal R(zgb-210::Tn10)Tet^S^ endA1 thi-1 ∆(hsdS-mcrB*)5 | Invitrogen |
| XL-10 GOLD | *endA1 glnV44 recA1 thi-1 gyrA96 relA1 lac Hte Δ(mcrA)183 Δ(mcrCB-hsdSMR-mrr)173 tet^R^ F'[proAB lacI^q^ZΔM15 Tn10(Tet^R^ Amy Cm^R^)*] | Agilent Technologies |
| pJC78 | pEXT20 coding for *ngt* and *agt,* IPTG inducible induction of NGT and α6GlcT | This study |
| pJC78 α6GlcT | pEXT20 coding for IPTG inducible NGT and α6GlcT. α6GlcT inactivated by mutating L7* | This study |
| pJC78 NGT K441A | pEXT20 coding for *ngt* and *gt,* IPTG inducible NGT and α6GlcT. NGT inactivated by mutating K441A | This study |
| pMKExpress | *sodCP, gfpmut3, aphA3, mob*  *A. pleuropneumoniae* complementation vector | (22) |
| pMK*ngt* | *ngt* cloned into EcoRI and SacI digested pMKExpress. Expressed under the control of the *A. pleuropneumoniae sodC* promoter. | This study |
| pMK*agt* | *agt* cloned into EcoRI and SacI digested pMKExpress. Expressed under the control of the *A. pleuropneumoniae sodC* promoter. | This study |
| pMLBADAtaC | pMLBAD(His10-AtaC-1866-2428)  arabinose inducible induction of AtaC | (24) |
| pEXT20 | Cloning and expression vector, pBR322 ori, IPTG inducible, Amp^R^ | (27) |
| pACYC184 | Cloning and expression vector, Tet^r^, Cm^r^, constitutive expression | New England Biolabs (UK) Ltd. |
| pJC1 | Constitutive expression of amino acid residues 23 to 163 of Cj0114 from *C. jejuni* NCTC 11168 with 12 C-terminal NAT glycosylation sequons and a C-terminus hexa-his tag. Cloned into BamHI and SpHI site within pACYC184 . | This study |
| pMKAcrANS | *acrA* cloned into the NotI and PstI digested pMKExpress. Expressed under the control of the *A. pleuropneumoniae* *sodC* promoter. Coding for AcrA lacking the first 22 amino acid residues of the PelB signal sequence encoded within pWA2 and C-terminus hexa-his tag. | This study |

**Table S2** Primers used in this study for reverse transcriptase analysis of RNA extracts from A. pleuropneumoniae HS143, complementation of ngt and agt mutants and construction of A. pleuropneumoniae deletion mutants.

| **Primer name** | **Sequence 5’-3’** | ***Target*** |
| --- | --- | --- |
| ngtFWDRT | TTTACCGTACGCATTCCACA | *ngt* |
| ngtREVRT | CCGTTGCTCTCACAAACTGA |  |
| RTagt-ngtFWD | AAGACAGGAGCCGAAGTTCA | *ngt to agt* |
| RTagt-ngtREV | GATTGATTTCCCGCCAATAA |  |
| ngt-rimOV2RTFWD | GTCGATAACCTTTCCGAGCA | *rimO to ngt* |
| ngt-rimOV2RTREV | AGTAATCGCCGTTGCCATAC |  |
| Mtn-rimO fwd | TCGCTTAATTCTTACGGAGCA | *mtn to rimO* |
| Mtn rimO rev | ACTAAATTCTTCGGACAGCCAAG |  |
| Mtn dsbA fwd | ACTTTGCCGATACCTGATTGT | *Mtn to dsbA* |
| Mtn dsbA rev | ACAATGACGACTATACTTACGGC |  |
| Slyx dsbAfwd | AAAGGCGCACAAGTCAGTAA | *Slyx to dsbA* |
| Slyx_dsbA rev | GCAGCATTACACGTTATGACCA |  |
| SodCFWDRT | AGCGGATAAGAGCTCAGTGG | *sodC* |
| SodCREVRT | CAGCTTGGGTTTTGGTGAAT |  |
| ngtagtCOMPFWD | TTTGAATTCCGAGCAAGAAGTGAAAGTCG | *ngt/ agt* |
| ngtagtCOMPREV | TTT GCGGCCGC CACCGATAGCCGTATTTCGT |  |
| agtCOMPFWD | AAACTGCAGATTAAATGCGTTCCTAAAAGAAAA | *agt* |
| agtCOMPREV | TTTGCGGCCGCTTAACTCCGACTATTCTCAAG |  |
| *ngt*-*agt*fwd | TTTTGAATTCCGAGCAAGAAGTGAAAGTCG | *ngt /agt* |
| *ngt-agt*rev | TTTTTGGTACCCACCGATAGCCGTATTTCGT |  |
| *1635_left* | CCCGTTTACAACAAGCGGTGGGTAAAACG | *ngt* deletion |
| *1635left_cat* | TCGGATCCGCGAATCCCACCGCTTGTTCAAACTCAATCTCATG | *ngt* deletion |
| *1635left_del* | TCCTGTCTTACACACCCACCGCTTGTTCAAACTCAATCTCATG | *ngt* deletion |
| *1635_right* | GGACTTGCATTACTTCCGGCACAATG | *ngt* deletion |
| *1635right_sac* | CCTCGAGCTTCACGCGTGTGTAAGACAGGAGCCGAAGTTCATG | *ngt* deletion |
| *1635right_del* | TTGAACAAGCGGTGGGTGTGTAAGACAGGAGCCGAAGTTCATG | *ngt* deletion |
| *1634_left* | CGATACAAGCGGTAGCATTGGGGCA | *agt* deletion |
| *1634left_cat* | TCGGATCCGCGAATCGCCGCCGGTGCAAGGTCTATTAAC | *agt* deletion |
| *1634left_del* | TACCCGTTTCACCCCGCCGCCGGTGCAAGGTCTATTAAC | *agt* deletion |
| *1634_right* | CCGCTTTCTTCACAAGCGGTTAAATCC | *agt* deletion |
| *1634right_sac* | CCTCGAGCTTCACGCGGGGTGAAACGGGTATATGGCAATATATTC | *agt* deletion |
| *1634right_del* | CCTTGCACCGGCGGCGGGGTGAAACGGGTATATGGCAATATATTC | *agt* deletion |
| *catsacB_for* | GATTCGCGGATCCGAGCTCTCTAAC | *catsacB*  cassette |
| *catsacB_rev* | GCGTGAAGCTCGAGGTATGGGATTC | *catsacB*  cassette |


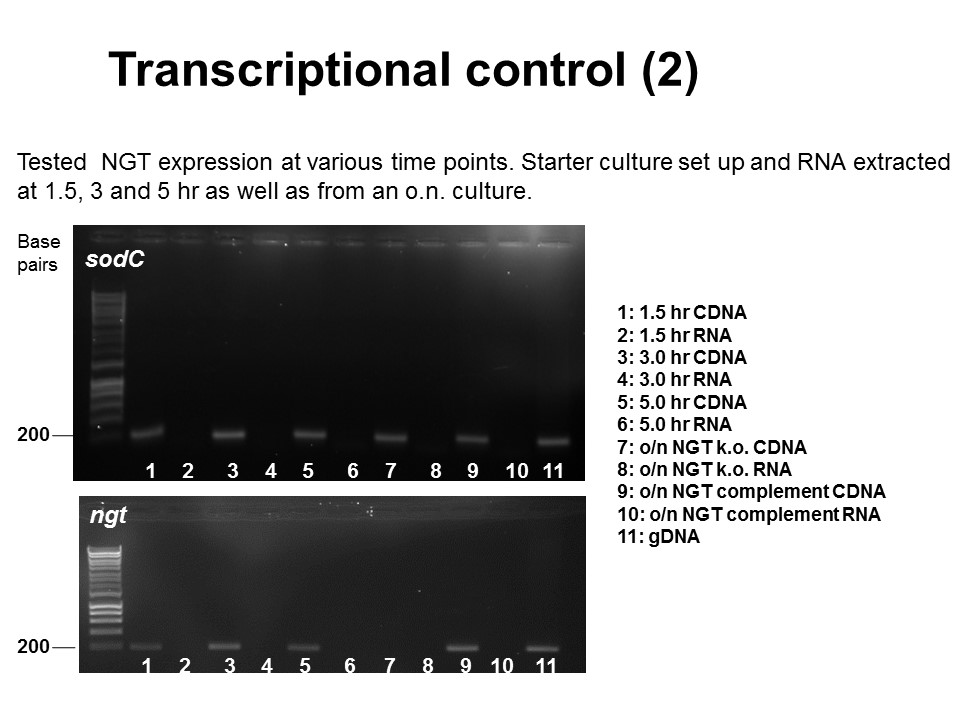


**Figure S1.** Expression analyses of ngt and sodC. Reverse transcriptase PCR results of RNA extracted from A. pleuropneumoniae serotype 15 at various time points and in various mutants. **1**, 1.5 hr cDNA template; **2**, 1.5 hr RNA template; **3**, 3.0 hr cDNA template; **4**, 3.0 hr RNA; **5**, 5.0 hr cDNA template; **6**, 5.0 hr RNA template; **7**, Δngt overnight culture cDNA template; **8**, Δngt overnight culture RNA template; **9**, Δngt complemented with pMKExpressNGT overnight culture cDNA template; **10**, Δngt complemented with pMKExpressNGT overnight culture RNA template; **11**, genomic DNA template (positive PCR control).

**
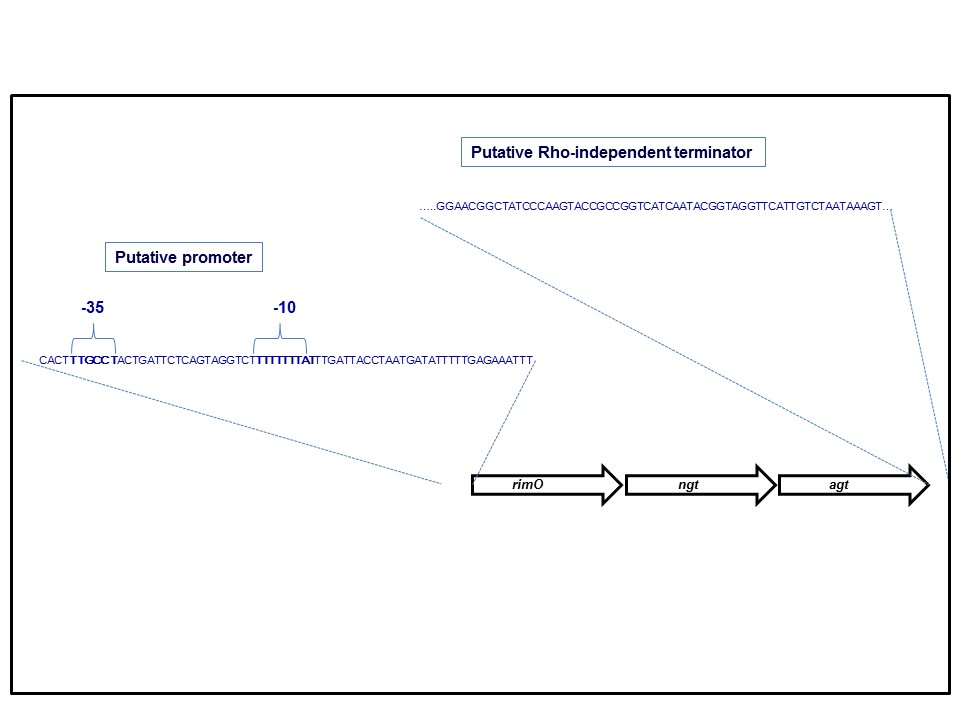
**

**Figure S2**. Predicted transcriptional promoter identified by BPROM and Rho-independent terminators identified by FindTerm bioinformatic analysis. V. Solovyev, A Salamov (2011) Automatic Annotation of Microbial Genomes and Metagenomic Sequences. In Metagenomics and its Applications in Agriculture, Biomedicine and Environmental Studies (Ed. R.W. Li), Nova Science Publishers, p. 61-78.
